# A Comprehensive Clinical Assessment of the LumiraDx International Normalized Ratio (INR) Assay for Point-of-Care Monitoring in Anticoagulation Therapy

**DOI:** 10.3390/diagnostics14232683

**Published:** 2024-11-28

**Authors:** Riffat Munir, Elise Schapkaitz, Lara Noble, Sakina Loonat, Melanie McCree, Nazeer Ali, Barry Jacobson, Wendy Susan Stevens, Lesley Erica Scott

**Affiliations:** 1 Wits Diagnostic Innovation Hub, University of the Witwatersrand, Johannesburg 2193, South Africa; 2 Haematology Department, University of the Witwatersrand and the National Health Laboratory Services, Charlotte Maxeke Johannesburg Academic Hospital, Johannesburg 2193, South Africa; 3 Haematology Department, University of the Witwatersrand and the National Health Laboratory Services, Chris Hani Baragwanath Academic Hospital, Johannesburg 1864, South Africa; 4 National Priority Program, National Health Laboratory Services, Johannesburg 2000, South Africa

**Keywords:** international normalized ratio, warfarin, LumiraDx Platform, point of care, standard of care, comparative study

## Abstract

Background: The International Normalized Ratio (INR) monitors anticoagulant treatment but relies on laboratory-based services. This could limit access to rapid monitoring and increase the diagnostic delay, both of which may be addressed by point-of-care testing (POCT). This study investigated the LumiraDx POC platform for INR monitoring. Methods: INR was measured on recalcified residual venous (*n* = 94) specimens from Chris Hani Baragwanath Hospital and capillary blood specimens (*n* = 254) from consenting enrolled participants at Charlotte Maxeke Johannesburg Academic Hospital Anticoagulation clinic, Johannesburg, South Africa. Standard-of-care (SOC) INR was measured on sodium-citrated venous blood using the Sysmex-CS2500 platform (Siemens Healthcare) and Neoplastin-R (Roche Diagnostics and Diagnostica Stago, Paris, France) within 2 h post-venipuncture. Within run, precision was measured using 2 LumiraDx control levels. The statistical agreement of paired INR measurements was also stratified by dosing decision. Results: The precision was within the manufacturer’s claim for controls (level 1%CV: 3.63, level 2%CV: 2.24). Accuracy analysis showed a moderate overall agreement compared to the SOC INR results with a correlation coefficient of 0.94 (95% Cl, (0.9267 to 0.9497)). The overall precision (ρ > 0.9) and accuracy (C_b_ = 0.9842) were good with an absolute bias of 0.07. The 95% confidence intervals for the slope and intercept did not include 1.00 and 0.00, respectively; however, the total calculated error was within the minimal acceptable limits. Conclusion: The LumiraDx INR Test showed a good performance compared to laboratory-based testing and provided opportunity for rapid and patient-centric care. Owing to an increasing positive bias for INR > 3.5, confirmation with laboratory INR measurements may be required.

## 1. Introduction

Warfarin is commonly prescribed for both preventive and therapeutic purposes in various conditions, including venous thromboembolism, heart valve replacement, and atrial fibrillation [1]. An overdose may lead to excessive bleeding, while insufficient levels can result in blood clots [1,2,3]. To ensure optimal anticoagulation, the regular monitoring of a patient’s International Normalized Ratio (INR) is crucial. The INR serves as the standard method of monitoring anticoagulant in patients on warfarin. The recommended INR for the treatment and prevention of deep-vein thrombosis, pulmonary embolism, and cardiac conditions, like atrial fibrillation and left ventricular systolic dysfunction, is 2.5 (with a range from 2.0 to 3.0). Individuals with mechanical prosthetic valves are typically advised a target INR of 3.0 (with a range from 2.5 to 3.5) [4,5,6,7].

The INR is conventionally determined by standard laboratory methods using citrated plasma obtained from venous blood [8]. Although these methods are considered the gold standard for monitoring anticoagulation, the process of obtaining results can be time consuming leading to delayed therapy. Point-of-care (POC) testing can provide a cost-effective and efficient alternative for INR monitoring and can be a valuable tool in a rural setting, where access to comprehensive healthcare facilities can be limited. POC testing enhances accessibility by reducing the need for long travel distances, allowing for timely diagnosis and treatment. Additionally, POC testing in consulting rooms offer significant advantages by providing immediate results, enabling rapid diagnosis and treatment, and improving patient compliance. It also facilitates the real-time monitoring of chronic conditions and reduces the need for multiple appointments, thereby lowering healthcare costs.

The LumiraDx Platform (LumiraDx UK Ltd., London, UK; www.lumiradx.com) is a POC system that comprises a portable LumiraDx Instrument and a LumiraDx Test Strip. Single or multiple instruments can be connected to the LumiraDx Connect Manager for extended functionality and configuration and results can be transmitted directly to the laboratory information system through its EHR Connect mode. The LumiraDx INR assay measures quantitative prothrombin time, which is converted to and reported as INR, directly from a fingerstick blood sample. When a blood sample is applied to the test strip, coagulation is activated by the recombinant thromboplastin, HemosIL, ReadiPlasTin. Prothrombin is converted to thrombin. Rhodamine, a peptide sequence, is cleaved and the substrate emits a fluorescent signal. The amount of signal detected over a specific time is converted via an algorithm into an INR [9]. The assay has a wide measurable INR range of 0.8–7.5 INR units with results available within 2 min. The hematocrit (HCT) is also assessed to ensure that specimens fall within the accepted 25–55% range.

The aim of this study was to assess the performance of the LumiraDx INR test in comparison to standard-of-care (SOC) results among patients using warfarin therapy. The evaluation involved utilizing citrated whole residual venous blood, an off-label specimen type, as well as directly applying capillary blood samples from individuals attending an anticoagulation clinic at the Charlotte Maxeke Johannesburg Academic Hospital (CMJAH).

## 2. Materials and Methods

### 2.1. Study Overview

Ethics approval for the use of residual samples was obtained from the University of the Witwatersrand Human Research Ethics Committee Medical (WITS HREC, Johannesburg, Gauteng, South Africa) under M1911201 to access residual clinical specimens. Ethics approval for the use of capillary blood from informed and consenting participants was obtained from WITS HREC under the clearance number M220657. Approval was also obtained from the South African Health Products Regulatory Authority (SAHPRA) with clearance number MD20220902 and the study was registered with the South African Clinical trials registry (Trial number: DOH-27-102022-5848).

**Figure 1:**
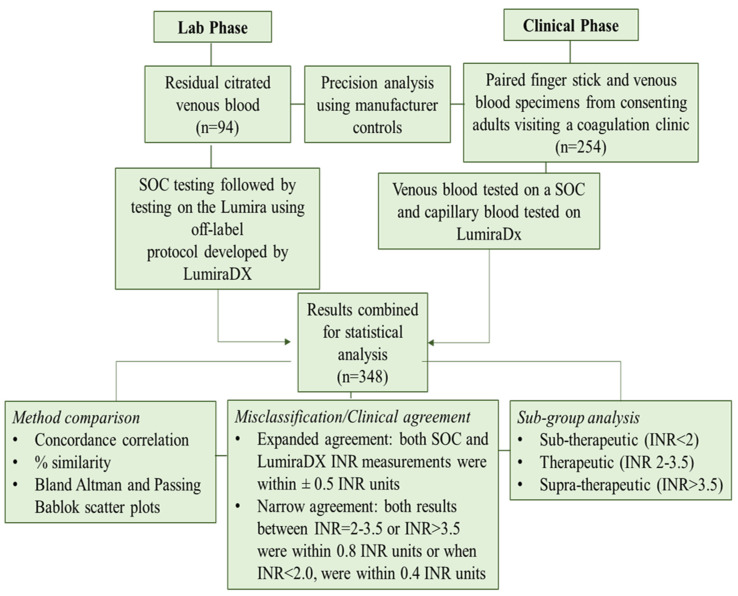
Overview of the study workflow.

The LumiraDx INR assay is intended to be used for testing capillary blood specimens from a finger stick. A rapid laboratory evaluation, using off-label recalcified plasma specimens as a proxy for the recommended finger stick protocol, was however performed initially to ensure analytical precision. This was followed by a clinical assessment in an anticoagulation clinic using finger stick capillary blood specimens. Results from both studies were combined for downstream analysis and compared to the standard-of-care (SOC) assay. Figure 1 provides an overview of the study workflow.

### 2.2. Patient Specimens

The laboratory evaluation was conducted using residual venous blood specimens (*n* = 100) at the SANAS accredited National Health Laboratory Services (NHLS) Haematology Laboratory at Chris Hani Baragwanath Academic Hospital Johannesburg, Gauteng, South Africa (CHBAH). Venous blood specimens from patients receiving warfarin therapy were collected in sodium citrate tubes (Becton-Dickinson, Oxford, UK) and referred for routine laboratory testing on the Sysmex CS2500 platform (Siemens Healthineers, EL, Forchheim, Germany) SOC assay. Once SOC testing was complete, the selected specimens, with varying levels of INR results, were recalcified according to the off-label protocol developed by LumiraDX and tested on the LumiraDx INR test. Briefly, 190 µL of plasma was recalcified with the addition of 10 µL CaCl_2_ solution and briefly mixed. Eight microliters were immediately tested on the LumiraDx INR assay using the instruments quality control mode.

For the clinical study, capillary blood specimens from 280 consenting adult patients receiving warfarin and visiting an anticoagulation clinic at Charlotte Maxeke Johannesburg Academic Hospital (CMJAH) were obtained by trained study staff over a period of 3 months. These were analyzed directly on the LumiraDX INR assay, according to the instrument’s standard operating procedures. Paired venous blood samples in citrate tubes were collected for SOC testing on the Stago STA R Max^®^ coagulation analyzers (Stago Diagnostica, Paris, France) using Neoplastin-R (Roche Diagnostics and Diagnostica Stago, Paris, France).

### 2.3. Precision Analysis (Within-Run and Between-Run Precision)

LumiraDx INR assay precision was determined by using 2 controls (low control: range of 1.0–1.6; and high control: range of 3.2–5.6) provided by the manufacturer. These were tested as 5 replicates over 5 days according to the EP15 protocol [10]. The mean and standard deviation (SD) were used to calculate the percentage coefficient of variance (%CV), which was compared to the manufacturer’s precision limits as well as Ricos biological variation acceptance criteria [11] www.westgard.com/biodatabase1.htm (accessed on 10 July 2024) and the European Federation of Clinical Chemistry and Laboratory Medicine (EFLM; https://biologicalvariation.eu/ (accessed on 10 July 2024)) [12] acceptable variation as outlined in Table 1. Ricos criteria and CLIA acceptance variation (https://www.govinfo.gov/content/pkg/FR-2022-07-11/pdf/2022-14513.pdf (accessed on 10 July 2024)) [13] were used to compare the %Bias and total analytical error, respectively.

### 2.4. Method Comparison

The LumiraDx INR Test was compared to the SOC results. Concordance correlation was applied to determine the degree to which data pairs align along the 45° line through the origin (measured by the correlation coefficient Pc) and provide an overall measure of precision (P, Pearson) and accuracy (Cb, bias correction factor) [14]. Overall agreement was considered poor for Pc < 0.9, moderate for Pc = 0.9–0.95, substantial for Pc = 0.95–0.99 and almost perfect for Pc > 0.99 [15]. The Bland–Altman [16] and percentage similarity [17] tools were applied as measures of agreement between the LumiraDx and SOC INR. The slopes and intercepts from the Passing–Bablok regression scatter plots were also evaluated to further assess the performance of the LumiraDx INR test. The results were deemed acceptable if the 95% confidence intervals for the slope included 1.00 and the intercept included 0.00.

Using previously defined criteria for the analysis of POC INR analyzers, the INR results were assessed overall and were further divided for sub-analysis into the following categories: sub-therapeutic (INR < 2.0), therapeutic (INR = 2.0–3.5), and supra-therapeutic (INR > 3.5) [7,18].

The clinically relevant agreement [6] was assessed by measuring discrepant INR measurements between SOC and LumiraDx, which would result in different warfarin dosages. This was measured using misclassification based on the SOC result across the three dosage categories. While not a clinically validated method, clinical agreement was additionally assessed by published criteria used for expanded and narrow clinical agreement [7,19]. Expanded agreement was achieved when both (SOC and LumiraDX) INR measurements were within ±0.5 INR units of each other, and narrow agreement was considered when both results between INR = 2–3.5 or INR > 3.5 were within 0.8 INR units or when INR < 2.0 and were within 0.4 INR units

### 2.5. Data and Statistical Analysis

Clinical study data were collected and managed using Research Electronic Data Capture (REDCap) electronic data capture tools [20,21]. Laboratory data were recorded on data collection sheets. Both clinical and laboratory data were combined and transferred to Microsoft^®^ Excel (Redmond, WA, USA) for analysis. Statistical analysis was performed using the MedCalc software package version 22.021 (Ostend, West-Vlaanderen, Belgium).

## 3. Results

During the laboratory phase of the study, six specimens were disregarded due to instrument errors. In the clinical phase, among the 280 participants initially enrolled, 26 were excluded for the following reasons: 18 due to HCT out of range or insufficient specimen errors, 5 due to SOC assay values exceeding the LumiraDx INR limit of detection, and 3 due to the unavailability of SOC results. The male:female ratio of the clinical study population was 1:1.6, with atrial fibrillation, valvular heart disease, and deep venous thromboembolism being the most common indications for anticoagulation.

**Figure 2:**
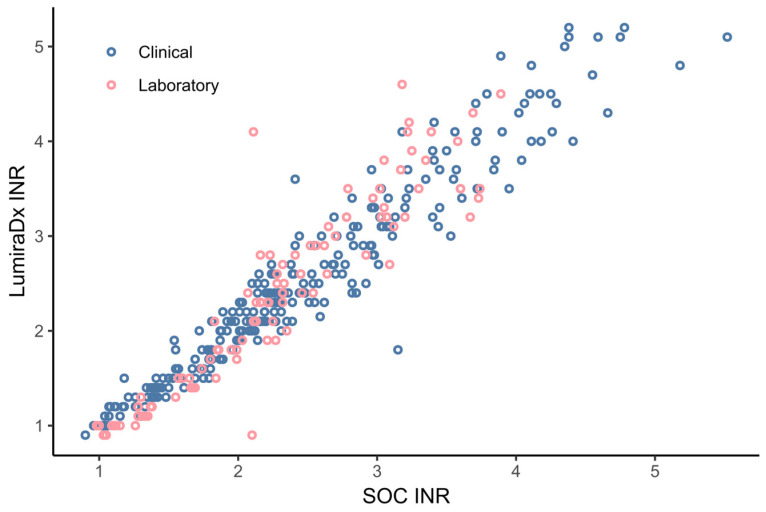
Overlay scatter plot of the laboratory and clinical datasets.

The overlap between the two datasets was determined by an overlay scatter plot (Figure 2), which showed that both sets had similar attributes/spread of INR values. The results from both laboratory and clinical phase data were therefore combined (*n* = 348 paired results) for method comparative analyses. The specimens tested across both phases displayed an overall INR range from 0.9 to 5.52 on the standard-of-care test.

### 3.1. Precision and Accuracy Analysis

The results for both within- and between-run imprecision of both low and high manufacturer-provided controls are presented in Table 2. The results obtained are within the manufacturer’s claims, as well as meeting the acceptance criteria outlined by EFLM, CLIA, and Ricos biological variation, as shown in Table 1. Minimal variability was noted with the low control (expected range: 1.0–1.6), while increased variability was observed for the high control (expected range: 3.2–5.6). However, it is important to note that no results were reported outside the expected ranges. The calculated total analytical error was within the optimum allowable error and therefore acceptable.

### 3.2. Method Comparison Analysis

Method comparison was carried out with a total of 348 paired results. This included 96 specimens tested during the laboratory phase and 254 results from the clinical phase. An overall error rate of 6% (24/380) was observed across both phases. Table 3 summarizes the results obtained from data collected across both phases and includes concordance correlation, Bland–Altman, and percentage similarity analyses.

#### 3.2.1. Overall Result Analysis

**Figure 3:**
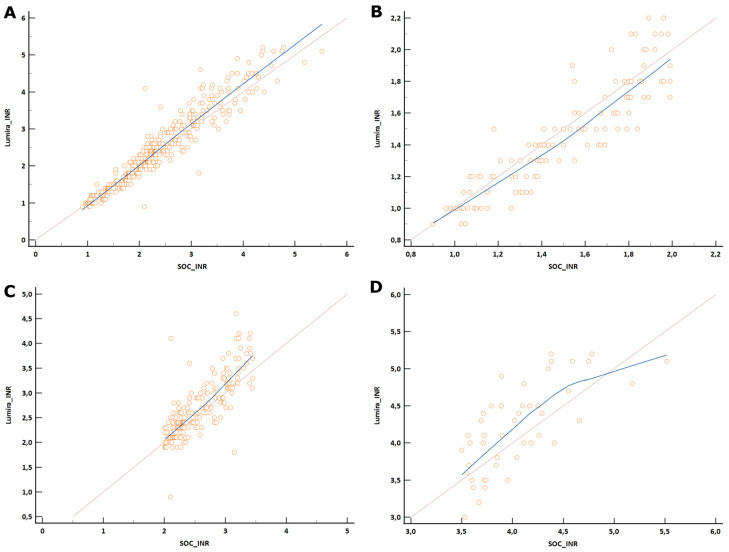
Concordance correlation of laboratory INR (SOC) and Lumira POC device INR for all ranges (**A**), sub-therapeutic range (**B**), therapeutic range (**C**), and supra-therapeutic range (**D**). An overall moderate agreement was observed across the entire data range (**A**) with a lower agreement noticed in the sub-categories (**B**–**D**). Orange line represents a central reference and the blue line represents best fit for the data points (regression line). INR, international normalized ratio; SOC, standard of care.

**Figure 4:**
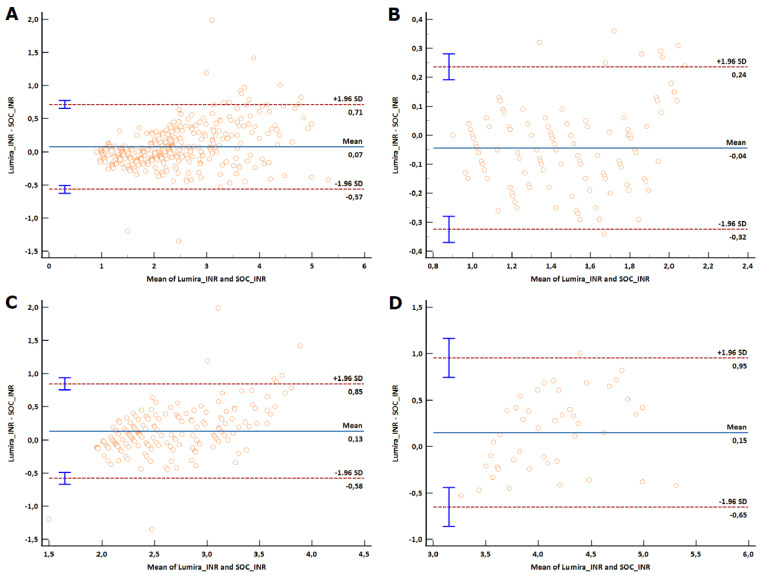
Bland–Altman difference plot comparing the laboratory INR (SOC) and Lumira POC device INR for all ranges (**A**), sub-therapeutic range (**B**), therapeutic range (**C**), and supra-therapeutic range (**D**). A small absolute bias was seen across all analysis with a random scatter, suggesting minor deviation from expected values. Blue lines indicate 95% confidence interval for upper and lower limits. INR, international normalized ratio; SOC, standard of care.

The overall mean obtained with SOC results (mean INR of 2.42 ± 0.89) was slightly lower than the LumiraDx INR mean (mean INR of 2.49 ± 1.02). The difference observed, however, was found to be non-significant (*p* = 0.3227). The LumiraDx INR assay showed a moderate overall agreement compared to the SOC INR results with a small absolute bias as noticed from Bland–Altman analysis (Table 3, Figure 3A and Figure 4A). The Passing–Bablok regression analysis indicated that 95% confidence intervals for the slope and intercept excluded 1.00 and 0.00, respectively (Table 4). The total calculated error was 10.9 and within the minimal acceptable limits.

#### 3.2.2. Sub-Therapeutic Category Analysis

A lower correlation was noticed between SOC and LumiraDx INR (Table 3, Figure 3B); however, a very good similarity of 99% was observed with an acceptable variation. The mean bias was notably small at −0.04, as evidenced by the Bland–Altman plot suggesting a minor but consistent deviation from the expected values. While a few outliers were observed, 95% of the values fell within the 2-standard deviation of the mean difference (Figure 4B). The 95% confidence intervals for both the slope and intercept included 1.00 and 0.00, respectively (Table 4), and the total calculated error was within the minimal acceptable limits (Table 3).

#### 3.2.3. Therapeutic Category Analysis

The mean bias between the SOC and LumiraDx INR was small at 0.13 (Figure 4C), with a low standard deviation of 0.37. While the agreement noticed was low (Figure 3C, Table 3), the similarity between the measurements was good at 102%. The slope and intercept values were 1.4 and −0.9, with the 95% confidence intervals excluding 1.00 and 0.00 (Table 4), respectively. The total calculated error remained within the acceptable limits (Table 3).

#### 3.2.4. Supra-Therapeutic Category Analysis

In the supra-therapeutic category, a low agreement was observed (Table 3, Figure 3D); the absolute bias however remained small at 0.15 with an acceptable SD of 0.41 (Table 3, Figure 4D). The percentage similarity was good at 101%. The 95% confidence interval did not include 0.00 for intercept and 1.00 for slope (Table 4); however, the total calculated error was within the minimal acceptable limits (Table 3).

#### 3.2.5. Clinically Relevant Agreement

Table 5 summarizes the clinically relevant agreement between paired INR readings obtained from both the LumiraDx and the Laboratory SOC. Overall, 91% of dual INR measurements met the expanded agreement criteria, while 97% met the narrow agreement criteria. Sub-category analysis revealed lower agreement levels, particularly in the supra-therapeutic range when assessed using the expanded criteria.

The further analysis of results that did not meet either the expanded or the narrow acceptance criteria revealed that 15 specimens (Table 6) would have required warfarin dose adjustments [6]. Of these, eight (shaded grey) were from the laboratory validation phase, while seven were from the clinical phase of the study. Most of these specimens (13 out of 15) were in the supra-therapeutic category, with only 7 falling within this range during the clinical evaluation phase. This highlights the potential need to re-test patients using a SOC assay for results falling in the supra-therapeutic category, as measured by the LumiraDx.

## 4. Discussion

This study provides a comprehensive evaluation of the LumiraDx INR assay as a POC tool for monitoring anticoagulation therapy in patients undergoing warfarin treatment. Due to the release of thromboplastin as a result of the finger stick injury, the validation of the LumiraDx INR assay compared to the standard venesection is critical if one is to use this method clinically. The LumiraDx INR test was compared to SOC INR measurements, and the findings indicate that the LumiraDx INR assay demonstrates moderate to good agreement with the SOC results, highlighting its potential for use in clinical settings.

The precision analysis of the LumiraDx INR assay revealed that both within-run and between-run variabilities were within the manufacturer’s claims and met the acceptance criteria set by the European Federation of Clinical Chemistry and Laboratory Medicine (EFLM), Clinical Laboratory Improvement Amendments (CLIA), and Ricos biological variation guidelines. The LumiraDx INR assay exhibited minimal variability, particularly with the low control, and slightly increased variability with high control samples. However, all results remained within the expected ranges, suggesting that the LumiraDx INR assay provides accurate results across the tested control ranges. Similarly, Tait et al. [22] in their study on the performance analysis of the LumiraDx INR assay obtained an overall precision of <4%. This was, however, derived by direct application or via a capillary transfer pipette of paired blood samples.

The comparability of the LumiraDx INR Test was assessed by correlating the INR measurements of capillary and venous whole blood to the venous plasma samples measured by the SOC instruments. The method comparison analysis showed that the LumiraDx INR assay had a moderate overall agreement of 0.94 with SOC results, similar to that obtained by Tait et al. [22] across the INR range from 0.8 to 7.5. The Bland–Altman plot indicated an acceptable absolute bias with low variability, indicating a consistent relationship between the two testing methods.

Sub-category analysis showed varying degrees of agreement across different therapeutic ranges. For sub-therapeutic range, the LumiraDx INR assay exhibited excellent accuracy and precision, with a 99% similarity to SOC results. The Bland–Altman plot indicated a minor but consistent deviation from the expected values, with 95% of the values falling within 2-standard deviations of the mean difference. The minor bias observed indicates that the LumiraDx assay is highly reliable for detecting sub-therapeutic INR levels. The agreement of 0.7 was less robust in the therapeutic range. The slight bias and the percentage similarity between the assays suggest that, while the LumiraDx INR assay can effectively identify therapeutic INR levels, there may be occasional discrepancies that warrant careful interpretation. Additionally, the total calculated error remained within acceptable limits, supporting the utility of the LumiraDx INR assay for patients within the therapeutic range. The poorest agreement of 0.67 was observed in the supra-therapeutic range. Despite the lower agreement, the bias remained small, suggesting that the LumiraDx assay can still be useful for detecting supra-therapeutic levels but may require confirmation with SOC testing.

Proportional and systematic differences were observed between the LumiraDx and SOC methods in all categories except for the sub-therapeutic range. These discrepancies are likely attributable to differences in the types of instruments used (benchtop vs. point-of-care testing).

The clinically relevant agreement was 88% (307/348) overall, with 91% of paired INR measurements meeting the expanded agreement criteria and 97% meeting the narrow agreement criteria. Sub-category analysis revealed a lower clinical agreement in the supra-therapeutic range, which highlights the potential need for retesting patients with SOC methods when LumiraDx results fall in this category. Patients with elevated LumiraDx INR results in the supra-therapeutic range should be promptly informed and retested using SOC methods. This retesting is crucial for accurately confirming their INR levels, preventing potential mismanagement of anticoagulant therapy and adverse outcomes, and ensuring that appropriate therapeutic decisions are made. Additionally, follow-up appointments should be scheduled to discuss the results of the formal INR testing and to implement any necessary adjustments to their management plan. Importantly, 15 specimens required different warfarin dose adjustments based on discrepancies between the two methods, predominantly in the supra-therapeutic range, underscoring the need for rigorous follow-up in such cases.

The study has several limitations. First, it did not assess lot-to-lot variation, linearity, or potential interference from certain medications, which may introduce variability and affect result reliability across a broader range of INR values. Second, differences in hematocrit levels among patients could have influenced INR measurements, as hematocrit impacts the accuracy of INR POC devices [23]. Third, the number of samples with INR values in the supra-therapeutic range was limited compared to those in the sub-therapeutic and therapeutic ranges, reducing the study’s statistical power at higher INR values. Fourth, while this study was conducted in a controlled hospital environment, stress testing on the device under varying temperatures or humidity was not performed, limiting insights into device performance in real-world conditions. Finally, although healthcare staff received training on the Lumira Dx INR assay, proper technique in capillary blood sampling and testing remains crucial for reliable outcomes. These limitations highlight areas for future investigation and emphasize the need for caution in interpreting the study’s findings.

## 5. Conclusions

The LumiraDx INR assay provides a POC testing method for monitoring warfarin therapy, with a good overall precision and accuracy compared to SOC methods. Its use can enhance patient care by providing timely results, reducing the turnaround time for INR monitoring, and potentially improving anticoagulation management. While the LumiraDx performs well for INR values below 3.5, caution is advised when interpreting results in the supra-therapeutic range (INR > 3.5), where confirmation with SOC testing may be necessary. With regards to usability, the LumiraDx INR assay has advantages over the laboratory-based SOC INR assay, for its use at point of care, its ease of use, and wide reportable range. In addition, the assay requires a smaller volume of blood and results are available within 2 min, which can be transmitted to the lab information system. Overall, the LumiraDx INR assay represents a valuable tool in the management of patients requiring anticoagulation therapy.

## Figures and Tables

**Table 1:** Precision limits and allowable error used in this study.

Analyte	Manufacturer Precision Claim		Westgard *, EFLM ^+^ and CLIA ^#^				
			Biological Variation		Allowable Bias (%)	Optimal Total Allowable Error	Minimal Total Allowable Error
International Normalised Ratio (INR)	Level 1 Control	Level 2 Control	CV_I_	CV_G_			
	6.5	6.5	4 *	6.8 *	2.0 *^+^	5.3 *^+^	15 ^#^

* Ricos Criteria; ^+^ EFLM; ^#^ CLIA, CVI intra-variability; CVG inter-variability.

**Table 2:** Measure of precision of the LumiraDx INR tested on the manufacturer control material.

Manufacturer claims	Control 1	Mean	1.25
		SD	0.081
		CV%	6.5
	Control 2	Mean	4.78
		SD	0.31
		CV%	6.5
	Capillary blood	CV%	3.73
Within run variability (CVi)	Control 1	Mean	1.2
		SD	0.00
		CV%	0.00
	Control 2	Mean	4.11
		SD	0.17
		CV%	4.01
Between run variability (CVg)	Control 1	Mean	1.2
		SD	0.00
		CV%	0.00
	Control 2	Mean	4.12
		SD	0.22
		CV%	5.31
%Bias	Control 1		4
	Control 2		13.81
Total analytical error (TAE)	Control 1		4
	Control 2		5.05
Westgard *, EFLM ^+^, CLIA ^#^	Optimal Allowable Error		5.3 *^+^
	Minimal Allowable Error		15 ^#^
Acceptable (Y or N)			Yes

* Ricos Criteria; ^+^ EFLM; ^#^ CLIA.

**Table 3:** Method comparison analysis for the overall dataset and clinically relevant sub-groups.

Category	Concordance Correlation				Bland-Altman		Percentage Similarity			Total Error			
	Correlation Coefficient (95%Cl)	Pearson ρ (Precision)	Bias Correction Factor C_b_ (Accuracy)	Agreement Score	Bias	SD of Bias	Mean % Similarity	% SD	%CV	Calculated Mean % Bias	Calculated Total Analytical Error (TAE)	Optimal TAE/Minimal TAE (Westgard ^*^ EFLM ^+^ and CLIA ^#^)	Acceptable
Overall (venous and capillary) (*n* = 348)	0.94 (0.93 to 0.95)	0.95	0.99	moderate	0.07	0.33	101.05	6.36	6.29	2.11	10.87	5.3 ^*+^/15 ^#^	yes
Sub-therapeutic (*n* = 117)	0.89 (0.84–0.92)	0.90	0.99	poor	−0.04	0.14	98.54	4.6	4.69	−2.91	5.85		yes
Therapeutic (*n* = 185)	0.73 (0.66–0.78)	0.79	0.91	poor	0.13	0.37	102.41	7.1	6.92	4.83	13.59		yes
Supra-therapeutic (*n* = 46)	0.67 (0.49 to 0.79)	0.72	0.93	poor	0.15	0.41	101.9	5.1	5.01	3.26	12.02		yes
clinical capillary FS (*n* = 254)	0.96 (0.94 to 0.96)	0.96	0.99	substantial	0.06	0.3	101.13	5.1	5.05	2.26	11.02		yes
Lab residual venous (*n* = 94)	0.89 (0.84 to 0.92)	0.92	0.96	poor	0.09	0.4	100.82	8.9	8.87	1.75	10.51		yes

* Ricos Criteria; ^+^ EFLM; ^#^ CLIA.

**Table 4:** Passing–Bablok analysis between the SOC and LumiraDx INR.

Parameter	*n*	Intercept	Slope	Comment
Overall INR data range	348	−0.28 (−0.363 to −0.205)	1.15 (1.111 to 1.186)	Systematic and proportional difference
Sub-therapeutic INR data range	117	−0.11 (−0.266 to 0.009737)	1.04 (0.9524 to 1.154)	_
Therapeutic INR data range	185	−0.9 (−1.1784 to −0.6165)	1.4 (1.2795 to 1.5286)	Systematic and proportional difference
Supra-therapeutic INR data range	46	−0.22 (−4.16 to −0.32)	1.57 (1.11 to 2.08)	Systematic and proportional difference

**Table 5:** Percentage agreement of paired INR measurements (LumiraDx INR and SOC) using clinically relevant agreement criteria.

Classification	*n*	Clinical Agreement	Agreement Based on Expanded Criteria	Agreement Based on Narrow Criteria
Overall	348	88% (307/348)	91.1% (317/348)	97.13% (338/348)
Sub-therapeutic	117	91% (106/117)	100% (117/117)	100% (117/117)
Therapeutic	185	86% (160/185)	89.7% (166/185)	95.68% (177/185)
Supra-therapeutic	46	81% (37/46)	73.91 (34/44)	95.7% (44/46)

**Table 6:** List of specimens (*n* = 15) where warfarin dosage categories differ between the two testing methods. Grey shaded specimens were from the laboratory validation phase and unshaded were from the clinical phase of the study.

SOC_INR	Lumira_INR	Difference	Dose Adjustment Needed
2.10	0.90	−1.2	Warfarin dosage increased by ~20% for LumiraDx
2.11	4.10	1.99	No change on SOC. Omit one dose and decrease weekly dose by ~10–20% on LumiraDx
3.18	4.60	1.42	No change on SOC. Omit one dose and decrease weekly dose by ~10–20% on LumiraDx
3.22	4.10	0.88	No change on SOC. Omit one dose and decrease weekly dose by ~10–20% on LumiraDx
3.23	4.20	0.97	No change on SOC. Omit one dose and decrease weekly dose by ~10–20% on LumiraDx
3.39	4.10	0.71	No change on SOC. Omit one dose and decrease weekly dose by ~10–20% on LumiraDx
3.69	4.30	0.61	No change on SOC. Omit one dose and decrease weekly dose by ~10–20% on LumiraDx
3.89	4.50	0.61	No change on SOC. Omit one dose and decrease weekly dose by ~10–20% on LumiraDx
3.15	1.8	−1.35	No change on SOC. Increase weekly dose by ~10% on LumiraDX
3.18	4.1	0.92	No change on SOC. Omit one dose and decrease weekly dose by ~10–20% on LumiraDx
3.41	4.2	0.79	No change on SOC. Omit one dose and decrease weekly dose by ~10–20% on LumiraDx
3.56	4.1	0.54	No change on SOC. Omit one dose and decrease weekly dose by ~10–20% on LumiraDx
3.71	4.4	0.69	No change on SOC. Omit one dose and decrease weekly dose by ~10–20% on LumiraDx
3.79	4.5	0.71	No change on SOC. Omit one dose and decrease weekly dose by ~10–20% on LumiraDx
3.89	4.9	1.01	No change on SOC. Omit one dose and decrease weekly dose by ~10–20% on LumiraDx

## Data Availability

The data presented in this study are available upon request from the contact author.
